# Frontline workers: Mediators of mucosal immunity in community acquired pneumonia and COVID-19

**DOI:** 10.3389/fimmu.2022.983550

**Published:** 2022-09-23

**Authors:** Priyanka S. Hastak, Christopher R. Andersen, Anthony D. Kelleher, Sarah C. Sasson

**Affiliations:** ^1^ The Kirby Institute, Immunovirology and Pathogenesis Program, University of New South Wales, Sydney, NSW, Australia; ^2^ Intensive Care Unit, Royal North Shore Hospital, Sydney, NSW, Australia; ^3^ Critical Care and Trauma Division, The George Institute for Global Health, Sydney, NSW, Australia

**Keywords:** lung, mucosal immunity, pneumonia, human, COVID-19

## Abstract

The current COVID-19 pandemic has highlighted a need to further understand lung mucosal immunity to reduce the burden of community acquired pneumonia, including that caused by the SARS-CoV-2 virus. Local mucosal immunity provides the first line of defence against respiratory pathogens, however very little is known about the mechanisms involved, with a majority of literature on respiratory infections based on the examination of peripheral blood. The mortality for severe community acquired pneumonia has been rising annually, even prior to the current pandemic, highlighting a significant need to increase knowledge, understanding and research in this field. In this review we profile key mediators of lung mucosal immunity, the dysfunction that occurs in the diseased lung microenvironment including the imbalance of inflammatory mediators and dysbiosis of the local microbiome. A greater understanding of lung tissue-based immunity may lead to improved diagnostic and prognostic procedures and novel treatment strategies aimed at reducing the disease burden of community acquired pneumonia, avoiding the systemic manifestations of infection and excess morbidity and mortality.

## Introduction

Pneumonia is a pathogen driven process of the lower respiratory tract that results in alveolar inflammation and impaired lung function (1). Symptomology includes fever, cough, dyspnoea, and chest pains. Community acquired pneumonia (CAP) is diagnosed in non-hospitalised patients or within 48 hours of hospital admission and is defined based on the clinical history and examination, biomarker evidence of infection as well radiological changes of a new infiltrate on the chest radiograph. At the more severe end of the spectrum CAP can lead to respiratory failure and sepsis (2) with associated extra-pulmonary organ failure. 10% of hospitalised CAP patients require critical care admission and mechanical ventilation. Until recently, the most common aetiological agents of CAP were bacterial in adults, although the current COVID-19 pandemic has demonstrated the high morbidity and mortality that can result from viral aetiologies (3).

In healthy individuals, the lungs are in continual contact with a plethora of inhaled bacteria and viruses, as well as host to their own commensal microbiome and virome. To maintain homeostasis, immunosurveillance of healthy lungs is required to identify potential pathogens and provide defence from these without long-term lung injury or inflammation (4). A complex network of tissue resident and infiltrating innate, innate-like and adaptive inflammatory immune cells, namely alveolar macrophages, dendritic cells, neutrophils, Mucosal Associated Invariant T (MAIT) cells and CD4^+^ and CD8^+^ T cells, are essential for surveillance of the external environment and pathogen clearance in the lungs (5). In this review we explore the current understanding of key mediators of localised lung immunity, including the dysfunction associated with diseased lung and the associated microbiome. While historically translational immunology has been studied primarily from the peripheral blood, an increased understanding of lung tissue-based immunity has the potential lead to improved diagnostic and prognostic procedures and novel therapeutic strategies aimed at reducing both pathogen-driven effects as well as aberrant host responses, both of which contribute to excess morbidity and mortality.

## Epidemiology of CAP

There is a significant burden of CAP globally with over 6 million people estimated to have died from COVID-19 by mid 2022. Pre-pandemic data attributes 3 million deaths per year due to CAP. Worldwide, the mortality rate of severe CAP has increased by 9% over the past three decades with 1.3 million deaths annually in adults >70 years, according to the World Health Organization (6, 7).

The overall incidence of CAP in Australia is 25 per 10,000 people, increasing with age to 319 and 660 per 10,000 for the 65-75 years and >75 years age groups, respectively (8). In the USA the incidence of CAP ranges from 25 cases per 10,000 to 106 per 10,000 people >65 years. Notably the incidence was reportedly higher in older CAP patients (9). The rising incidence of CAP with age has been replicated in South America, Asia, and the United Kingdom (8–10). Overall, the significant burden of disease from CAP has been illustrated globally, with data repeatedly demonstrating patients >65 years of age are at the highest risk of severe disease.

The risk of CAP also increases with premorbid clinical conditions, lifestyle, environmental factors, and use of certain medications. A systematic review of 29 observational studies concluded that clinical conditions that definitively increase the risk of CAP include chronic bronchitis, chronic obstructive pulmonary disease (COPD) and asthma. Orodental and/or periodontal disease and a poor nutritional status (e.g. malnutrition, hypoalbuminemia) were also identified as risk factors (11). Lifestyle factors such as smoking were a clear risk although a definitive conclusion was not made for alcohol use. Environmental factors in the home, workplace or local environment such as exposure to a variety of substances including dusts, fumes, solvents and asbestos also increased the risk of CAP. Immunosuppressive therapies including oral steroids and oesophageal reflux therapies such as proton pump inhibitors and H2 receptor antagonists were also determined to be risk factors for CAP (11).

Co-infections and secondary bacterial infections are known to be associated with poor patient prognosis and CAP. There is a complex interaction between respiratory pathogens such as influenza and bacterial co-infections like *S. pneumoniae*, that leads to more severe disease (12). Influenza A has been associated with an increase in the colonization and transmission of secondary bacterial infections, including *S. pneumoniae* and *S. aureus* possibly through a mechanism of viral priming (13, 14). Bacterial pneumococcal colonization was 100,000X times higher in the nasopharynx in patients with influenza A infection compared to those without influenza. Viral influenza impacts bacterial colonization and replication up to 6 months post infection (15). Moreover, the duration of bacterial infection rises by 2-5 times in these patients (14).

During the 2009 USA H1N1 pandemic, 30-50% of hospitalized CAP patients harboured pneumococcal pneumoniae co-infection in their lung (16). Another report demonstrated that 38% of H1N1 associated deaths with no comorbidities harboured *Streptococcus* co-infections (17). Other respiratory pathogens, like respiratory syncytial virus (RSV) which is known to cause acute respiratory tract infection in the lungs and respiratory tract, were typically associated with concurrent or secondary bacterial infections with *S. pneumonia, H. influenza* and methicillin resistant *S. aureus* (18). These infections were associated with higher risk of CAP, ICU admissions and higher mortality (19).

There are varying reports of co-infections and superinfections identified in COVID-19 pneumonia patients (20). Initial studies indicated that there was less frequency of co-infections or secondary infections during SARS-CoV-2 infection compared to other respiratory infections like influenza (21). However, more recent studies are contradictory, one of which was on 257 adults and children with COVID-19, where 94.2% of the cohort carried one or more respiratory pathogen, with high rates of *S. pneumoniae*, *K. pneumoniae* and *H. influenza*. These coinfections occurred 1-4 days after SARS-CoV-2 infection (22). Notably, patients with severe SARS-CoV-2 pneumonia had 10 times higher chance of bacterial superinfections and fungal infections in the lungs (23). A study in Wuhan identified a variety of co-infections from COVID-19 bronchoalveolar lavage (BALs), sputum, nasal swabs and blood. Although they found *M. pneumoniae* was predominant in sputum, other pathogens such as *E. coli, A. baumanii* and *P.aeruginosa* and *Candida* spp., were found in both BAL and blood (24). Notably, more than 8% of these infections developed during hospitalization and resulted in severe pneumonia (24). In summary, the complex and dynamic relationship between respiratory pathogens and co-infections or super infections is crucial to investigate to determine targets for improving clinical outcome in pneumonia patients.

## Biomarkers of CAP

The severity of CAP is routinely summarised using clinical scores, such as CURB65 (confusion, urea nitrogen, respiratory rate, blood pressure, age of 65 years) or the Pneumonia Severity Index (PSI) that provide guidance for determining 30-day mortality and management of CAP patients (25). Inflammatory biomarkers have been found to be useful in diagnosis and monitoring of CAP. These inflammatory markers include C-reactive protein (CRP), and procalcitonin (PCT).

CRP typically induced by IL-6, IL-1β and TNF-α CRP has been identified in bacterial pneumonia patients as early as the 1930s (26). CRP in healthy individuals is generally ≤ 5mg/L but in patients with pneumonia the levels can be >100mg/L (27). One study found high levels of CRP was associated with high sensitivity (100%) and specificity (90%) for patients with symptomatic pneumonia and correlating radiographic features (28). Although the increase in CRP levels was reported to be a useful indicator for diagnosis of pneumonia, there was no association of CRP with severity of illness or symptoms.

PCT is a proposed diagnostic marker for bacterial pneumonia leading to sepsis (29). In hospitalized CAP patients, the median serum PCT levels were reportedly lower in viral infections (0.09ng/ml) compared to typical bacterial infections (2.5ng/ml). The median PCT levels differed in typical (2.5ng/ml) and atypical bacteria (0.20ng/ml) in one cohort (30). The PCT threshold of 0.2ng/ml generated 80.9% sensitivity but only 51.6% specificity for classifying infections as bacterial. Although elevated median PCT indicated a high likelihood of bacterial infection, there was no clear threshold to distinguish viral and bacterial CAP infections (30). Despite this, PCT is often used in acute respiratory infections to rationalise and reduce antibiotic exposure (31).

Neutrophil to lymphocyte (NLR) ratio is a parameter that is typically used to determine inflammatory status and progress of disease (32). NLR has been widely used as an early prognosis marker for COVID-19 patients. It is also associated with increased risk of mortality during hospitalization (32). It is well established that neutrophils are activated in acute COVID-19 infections, and they are activated by inflammatory cytokines including IL-6, IL-8, TNF-α, GSF and IFN-γ (33). Lymphopenia is typically observed in respiratory sepsis, including that caused by COVID-19. Hence the range for LNR is set between 3 to 6, where high neutrophil count or neutrophilia and low lymphocyte count or lymphopenia is recorded (34). Reportedly COVID-19 patients had lower NLR at hospital admission, but for patients with severe COVID-19 the cut off was as high as 6.82 (32). This prognostic marker was valuable to distinguish poor patient outcome.

Overall, from a clinical perspective, these biomarkers may provide additional evidence for CAP and can help distinguish between different aetiologies, however they are imperfect and need to be applied within the context of the patient’s presenting history, examination, and other investigations

## Lung microenvironment

Several host defence mechanisms protect the lung microenvironment, these include: physical, chemical, biological, and adaptive. For instance, the pulmonary epithelium that comprises of the alveolar and the airway epithelium form a boundary from the outside environment and protect the lungs from pathogens and microbes. The airway epithelium is comprised of ciliated columnar cells and secretory cells including mucus secreting goblets, clara and serous cells that are connected to form barrier surface (35). The tight junctions are composed of occludins and adherins such as E-catherin which create semipermeable divisions between epithelial cells. The alveolar epithelium can be of two types: type I associated with gas exchange and type II that secrete surfactant lipids and proteins to maintain homeostasis (35).

In lower respiratory tract infections, the respiratory viruses or bacteria invade the muco-ciliary barrier, damage the airway epithelium, and consequently destroy the tight junctions to elevate epithelial permeability (36, 37). The secretory cells constitutively produce mucin molecules and several antimicrobial peptides (AMP) including cathelicidin and secretory leukocyte protease inhibitors, histones, surfactant proteins SP-A and SPD all essential for optimal lung function (37).

In healthy individuals, mucus is secreted in small amounts in the airways and is composed of mucins, and enzymes as well as several defence proteins. Mucins such as MUC5AC are released in the lower airway tract by secretory goblet cells and MUC5B is produced throughout the airways. MUC5B expression has been associated with inflammation and phagocytotic response in the lungs (38). In MUC5B knockout mice the production of IL-23 was reduced and associated with an increase in macrophage apoptosis (39). Moreover, there was low antibacterial response mounted in these knockout models. Hence MUC5B appeared to play a key role in the human airway to augment the mucosal barrier and particle clearance (39). The clearance of inhaled particle matter is facilitated through the muco-ciliary action of the airway epithelium as well as coughing. Initial symptoms of impaired mucus clearance are cough and dyspnea. Mucus and inflammatory exudates can be identified in chest radiographs (40).

The defence proteins present in the mucus include (i) annexin A2, that protects the lungs from pathogenic insults and foreign particles (ii) lumicin which is an extracellular matrix proteoglycan and (iii) keratin that has antimicrobial properties (41, 42). The hydration of mucus drastically affects these proteins as well as its viscosity and consequently determines how efficiently invading pathogens or toxins can be cleared by coughing. An optimal balance of mucus production in the respiratory airways is essential for healthy lung function (40).

## Cellular mediators of pulmonary immunity

### Alveolar macrophages

The lungs are populated with variety of macrophages that differ based on their origin and tissue residency. Macrophages are mononuclear phagocytes and within the lungs they are denoted as alveolar or interstitial macrophages. Alveolar macrophages (AM) reside in the alveolar space and represent the major phagocytic and antigen presenting cells (APC) in the respiratory tract (43, 44).

Recruited AM initially induce inflammation and later suppress it through multiple pathways, this involves converting from the inflammatory (M1) to anti-inflammatory (M2) macrophages, phagocytosis of apoptotic cells for neutrophil clearance, expression of cytokines, like IL-10 and IL-1R antagonists (45). A study demonstrated that AM from the murine lungs following influenza had defective NF-κB nuclear translocation to Toll-like receptor (TLR) ligation that resulted in reduced acute inflammation, chemokines (IL-10, MCP-1, MIP-1) and cytokines (IL-1β, IL-6, TNF-α) and neutrophil recruitment (34).

Unlike in mice, recent human studies have reported that AM can express M1 and M2 markers simultaneously, which has been coined as a “hybrid” AM phenotype. The categorization of polarized M1 and M2 phenotype has been mainly identified in mouse models or in *in-vitro* experiments of human macrophages (46). The hybrid M1/M2 phenotype was characterized in BALs, and majority of the AMs expressed surface markers CD206^high^ and CD86^high^ that is typically found in M2 and M1 phenotype respectively. The combination state of M1/M2 hybrid phenotype has been essential to facilitate rapid and adaptive responsiveness to respiratory pathogens in the lung mucosa (47).

Aging is a major factor that influences function and abundance of AM. Younger populations have a larger pool of AM compared to older populations (48). When transcriptomic analysis was conducted on lungs of old mice, they found differences in 3,545 genes associated with AM compared to young mice, with downregulation of cell expression pathways during influenza infection (49). Aging has also been linked to higher neutrophil accumulation in the lungs as a result of dysfunctional AM. This retention of neutrophils was associated with increased lung damage. Notably, aging lead to a reduction in both the number and function of AM even prior to influenza infection (49). Hence the self-renewal ability of AM decreased over time. This decline in AMs with age has been associated with higher mortality and pneumonia in older populations (49).

AM are essential for healthy lung function as they remove cellular debris and apoptotic cells. They also assist in bacterial clearance and initiate neutrophil recruitment and regulate cytokines including IL-10, TGF-β and surface receptors like CD200 associated with AM homeostasis (50). When AM were not present in the lungs of mice, there was severe lung dysfunction and respiratory failure (51, 52). The activation of AM in the lungs has been recently explored where AM had reduced responsiveness to IL-4-driven inflammation in the lungs compared to macrophages from other mucosal sites. When the AM were removed from the lungs, they were responsive to IL-4 *via* glycolysis, suggesting that that the lung microenvironment regulated the AM responsiveness (53). AM have specialised phenotypic, metabolic, and functional plasticity (34). A study analysed ~73 metabolites in influenza infected mice lungs and revealed that certain metabolites such as creatinine and phosphocreatine differed in their expression in naïve versus experienced AMs. When principal component analysis was conducted to compare the metabolites identified in the two types of AMs, there was a very large variance of 32% observed. This suggested that experienced AMs had altered metabolic activity in mice that were infected (46, 54, 55). Hence AM may have the ability to alter the metabolic activity during respiratory infections.

In inflamed lungs, AM are activated to express inflammatory mediators such as TNF-α, IL-1β, IL-6 and TLR-4 that facilitate the destruction of airway epithelium and loss of barrier functions. Consequently, an alveolar oedema occurs that is clinically denoted as Acute Lung Injury (ALI), a predisposition to acute respiratory distress syndrome (ARDS), which is a severe inflammatory condition of the lungs associated with 30% increase in mortality (56). Very little is understood about pathogen-specific alveolar inflammation, or indeed the drivers of ARDS and its resolution (57).

A common method to study alveolar macrophages and other local cellular mediators is through the study of BAL samples. BAL is a procedure that involves sterile saline being passed through the bronchoscope to wash the lung airways prior to aspiration and collection of the sample. A recent study approximated that the total AM in mice alveoli was ~4X10^6^, but BAL macrophages were estimated to be ~1x10^6^. BAL was reported to accesses macrophages that were loosely bound to the epithelium and represents a fraction (around 25%) of the macrophages present (58). BAL sampling is a common clinical procedure aiding diagnosis but also represents and under-utilised avenue. Future studies that include BAL sample analysis to study these macrophages and other immune cells represent an advancement on peripheral blood analysis in isolation, particularly regarding the assessment of the inflammatory microenvironment including tissue-resident cells in respiratory disease (59).

### Monocytes

Circulating monocytes are precursors of resident and inflammatory tissue macrophages (60). Peripheral blood monocytes are typically distinguished into two broader functional groups, classical and non-classical monocytes, based on the expression of CD14 and CD16 as well as the cytokine receptor CCR2 and fractalkine receptor CX3CR1 (61). In the lungs, migratory monocytes are found within the blood vessels and between the lung capillaries and alveoli. Lung monocytes differ from circulating monocytes in that they are heterogenous with distinct marker expression including CD11b, HLA- DR, CD11c, CX3CR1, CD62L and CD117 (62).

Classical monocytes are pro-inflammatory and produce soluble mediators including secretory leukocyte protease inhibitor (SPLI), TNFα, IL-1β, CCR-2, CCL-2 and Ly6c^+^. In mouse models, these classical monocytes have the ability to differentiate into monocyte derived dendritic cells and macrophages that bridge innate and adaptive immune responses. In processes such as pulmonary infection or inflammation, there is rapid recruitment of monocytes to tissues and associated differentiation (60). A study of peripheral blood in mice indicated that monocyte subsets were essential markers of disease progression in COPD and idiopathic pulmonary disease (63). Although monocytes are major contributors in triggering the immune response and driving inflammation, there a few studies interrogating how monocytes behave once they enter the lung tissue particularly in patients with pneumonia and/or ARDS (63). A recent study of COVID-19 BAL samples revealed that proinflammatory monocytes are recruited to the lungs and differentiate into alveolar macrophages. After differential gene expression analysis, two major groups were identified, with one of the groups expressing peripheral monocyte markers S100A8, FCN1 and CD14. Both groups had gene expression markers typically identified in M1 proinflammatory macrophages (64) and these monocyte derived AM have also been identified in other respiratory infections as well. Investigation of BAL samples from patients with *S. pneumonia* found a high prevalence of AM in the airways when the bacteria colonized in the nasopharynx. Also, the AM: monocyte ratio was 2.3 times higher in the lungs where bacterial colonization was present, compared to no colonization (65). A study on acute influenza infection in mice reported that one month post infection the number of macrophages increase in the lungs and these macrophages transcriptionally resembled monocytes (66). These monocytic-like AM cells expressed high levels of IL-6 that protected mice from co-infections and secondary infections. After 2 months of influenza infection these recruited AM develop a tissue-residing phenotype and as a result are unable to provide antimicrobial immunity (66). It may therefore be evident that monocyte-derived AM are key players for changes in the lungs post infection. How these AM-like monocytes influence homeostasis of the lungs requires further investigation.

### Dendritic cells

Dendritic cells (DCs) are antigen presenting cells (APCs) that represent an interface between innate and adaptive immune responses. Immature DCs interact with antigens, retaining them without presentation. Immature DCs reside in airways epithelium and pulmonary vessels but do not typically appear in the alveoli of healthy lungs. In the presence of antigen, immature DC are stimulated by TLRs or proinflammatory cytokines, and consequently upregulate CCR7 which facilitates the migration of DCs to the lymph nodes and simultaneously produces costimulatory molecules for efficient antigen presentation. DCs capture pathogen-derived antigen and process and present antigenic epitopes to naïve T cells *via* major histocompatibility complex (MHC). DCs facilitate viral clearance and produce type I IFN and cytokines such as IL-12 upon stimulation. They are able to convert to immunogenic DCs that can prime T cells to fight viral pathogens (67, 68).

Two DC phenotypes are essential for lung host defences: classical DC (cDCs) or myeloid DCs and plasmacytoid DCs (pDCs). The cDCs can be categorized as cDC1s that express CD141^+^ marker and cDC2s that expressed CD1c^+^ (69). They express several TLRs including TLR1, 2, 3, 4 allowing them to be activated by mycopeptides and viral RNA through the production of IL-2. The pDCs mainly express TLR7 and TLR9 facilitating response to bacterial DNA and viruses through the production of inflammatory cytokines e.g. IFN-α (70). In influenza A-associated pneumonia mice lungs, TLR7 and TLR9 stimulated the maturation of DCs (70). In murine RSV infection, TLR3-activated DCs mediate antiviral activity, where RSV replication led to rise in TLR3 protein expression on cell surface (71). When TLR3 protein was blocked it decreased the production of pro-inflammatory mediators IL-6, CXCL8 and CCL-5 within bronchial epithelial cells, but this increased after RV infection. Hence TLR-activated DCs may be vital for triggering the immune response during a viral infection both locally and systemically (72).

COVID-19 infection impacts the proportion and function of DCs and also inhibits rapid regeneration of these cells (73). When BALs from severe COVID-19 patients were assessed, they found abundance of cDC2s but very low prevalence of pDCs and cDC1s. These depleted number of pDCs and cDCs in the lungs were associated with downregulation of inflammatory chemokines CCR2 and CXCR3 (69, 74). Similarly, another study on severe COVID-19 BALs revealed lower levels of pDCs and mDCs compared to mild COVID-19 BALs (64). Not only is there a reduction in DC subsets but also in their ability to function during COVID-19 infection. For instance, antigen presentation was reportedly impaired in DCs during COVID-19 infection in the lungs and they failed to stimulate the proliferation of CD4^+^ and CD8^+^ T cells (75, 76). In addition to antigen presentation impairment, DCs lost their capacity to produce type I IFN, a key player in antiviral immunity (73). In the lungs of COVID-19 pneumonia, pDCs were unable to produce IFN-1β and the lack of IFN-1β production was linked to high severity and hospitalization in these patients (77). Interestingly, when severe COVID-19 pneumonia patients were administered IFN-1β combination therapy there was improved clinical outcome in these patients compared to patients without IFN- 1β therapy (77).

### MAIT cells

MAIT cells are present in blood and enriched at mucosal sites including the lower respiratory tract and intestines. In humans, these innate-like T cells typically express CD8, a semi-invariant Vαß 7.2-Jα33 T cell receptor (TCR) and CD161 and lack CCR7 (78). They can also express CD4 or be CD4^-^ CD8^-^ but these subsets are less abundant compared to CD8^+^ MAIT cells (79). Stimulated MAIT cells express high levels of IFNγ, TNFα, and several cytokine receptors IL-7R, IL-15R, IL-12R and IL-23R. MAIT cells can be categorised into MAIT-1 and MAIT-17 depending on the differential production of cytokines (80, 81).

MAIT cells play a crucial role in early immunity against infections in peripheral tissue. They can be activated *via* both TCR-dependent and independent pathways. In the former, MAIT cells recognise microbial riboflavin-based metabolised antigens presented by the MHC-like molecule, MRI. TCR-induced signalling is essential for activating MAIT cells by riboflavin-producing bacteria or yeast consequently leading to cell-mediated killing of these microbes. TCR-independent activation of MAIT cells occurs *via* cytokine signalling. Initial studies found cytokines IL-12 and IL-18 associated with MAIT cell production of IFN-γ and IL-17 as well as granzyme B (82). Subsequent studies have identified additional cytokines IL-15, IFNα/β and TFNα that activate MAIT cells (78, 83). MAIT cell activation is promoted by the TLR1, TLR2 and TLR6 pathogen recognition receptors in humans (80, 81). In mouse models of respiratory infections, MAIT cells recruit T cells to the lungs and promote inflammatory monocytic differentiation into dendritic cells (82).

MAIT cells are highly prevalent in humans and constitute 1-3% of T cells in the blood and higher percentages in mucosal sites. In contrast, these are rare in mice, representing less than 1% of the total T cells (84). Hence mouse models are not ideal for investigating into this cell type. In humans, MAIT cells were found to protect against colonisation of *S. pneumoniae* in the nasal passage. High levels of MAIT cells in the nasal mucosa and the production of TFN-α and IFN during *S. pneumoniae* infection could lead to recruitment or activation of neutrophils and monocytes in the lungs (85, 86). In a study of sputum samples from patients with mild CAP, a higher proportion of MAIT cells were found in CAP patients as compared to healthy controls. The abundance of MAIT cells was correlated to the production of IFN-α, IFN-γ as well as neutrophil abundance (87). In BAL samples, the presence of subset of CD103^+^ MAIT cells was associated with hypoxia in the inflammatory tissue as assessed by the high expression of hypoxia-induced factor (HIF-1) that is associated with increased IL-17 expression. This study also indicated that an increased proportion of MAIT cells in the local microenvironment improved clinical prognosis (88, 87).

In patients with severe COVID-19, MAIT cells are reduced in circulation while increased in the respiratory airways as determined by single cell RNA sequencing data (89), suggesting recruitment of cells from the circulation to the primary site of infection. Transcriptional analysis revealed MAIT cells released high levels of TNF-α but not IFNG or granzyme B. The MAIT cells had a CD69^+^ and CXCR3^-^ phenotype in both peripheral blood and the airways (89). This phenotype as well as high PD-1 levels were associated with poor patient survival. Interestingly, in patients deceased from COVID-19, there was an inverse correlation between MAIT cells and days since symptom onset, suggesting that a MAIT cell activation at disease onset may drive the immunopathology and be associated with poor patient outcome (90).

### Neutrophils

The recruitment of neutrophils into the lungs and promotion of localized inflammation involves macrophages, epithelial cells, monocytes, and other immune cells as well as cytokines and chemokines (91). Neutrophils are vital in phagocytosis and elimination of pathogens using reactive oxygen species, antimicrobial agents, and serine proteases (92). A study on *S. pneumoniae* infected mice revealed that during infection if the neutrophils reduced in the lungs, this had a negative effect on viral clearance (93, 94). Neutrophils are known to produce chromatin DNA associated with antimicrobial agents and enzymes, called as neutrophil extracellular traps (NETs). Their function is to eliminate virulence factors and act as antimicrobial agents thereby preventing dissemination of infection. During pneumonia, NET levels were found to be high in the lungs of both humans and mice (95). A report to investigate the role of NETs in ARDS in mice was conducted (96). Here a mice model of ARDS from methicillin resistant *S. aureus* and *Pseudomonas aeruginosa* was established and the results revealed that NETs increased with ARDS severity and was associated with high mortality (96). Additionally, excess production of NETs increased tissue damage and sepsis (96, 97). Neutrophil activation can lead to increase in release of neutrophil elastase which contributes to tissue damage (98). Neutrophil elastase also impacts lung extracellular matrix and alveolar gas exchange (99). Rise in neutrophil elastase levels has been correlated to pneumonia, ARDS and exacerbated chronic obstructive pulmonary disease. Notably, studies on BAL from mice infected by pneumococcal infection demonstrated rise in activated neutrophil and NET activity (95, 100, 101). A study of immune cells in BAL samples from patients with COVID-19 indicated high neutrophil count in severe cases compared to moderate cases (64). Overall, these reports indicate the importance of NETs in CAP, ARDS and COVID-19.

### NK cells

Natural killer (NK) cells are a heterogenous cell type with varied functions that differ depending on the local microenvironment. Their role and phenotype in the lungs differ from other tissue and blood. In the lungs, NK cells are associated with homeostasis and constitutes as high as 10-20% of lymphocytes (102). Human lung NK subsets typically have a CD56^+^CD16^+^ phenotype. Additional cell markers expressed by NK cells found in the lungs include, KIRs (killer cell immunoglobulin receptors) and CD57^+^ (103). During influenza infection NK cells were found to be activated *via* IFN-γ production that was crucial for viral clearance (104, 105). These cells are activated by other cytokines as well, namely, IL-12 and IL-2 and other type I IFN (102). In bacterial infections such as *K. pneumoniae*, lung NK cells promote host defences by releasing the cytokines IL-22 and IFN-γ (106). Hence a varied range of cytokines activate NK cells during a bacterial or viral infection.

It has been demonstrated that vaccines induce memory NK cells, for example, in influenza vaccinated mice. Here memory NK cells have the ability to inhibit secondary influenza infections (107). In human lungs, the NK cells can respond quickly upon exposure to *ex-vivo* influenza infection of lung explants (108). Interestingly, the function of NK cells in the lungs was further investigated, where *K. pneumonia* pre-infection increased the likelihood of influenza-induced acute lung injury by blocking lung NK cell proliferation (109). Recently the role of NK cells in antiviral SARS-CoV-2 activity was identified in blood and explored in BALs. Pulmonary NK cell dysfunction was involved in progression of fibrotic lung disease in patients with severe COVID-19 (110). NK cells were activated with higher IFN-γ production in BALs from patients with severe COVID-19, compared to those with moderate disease (64). COVID-19 BALs expressed high number of cytokines, such as CCL3, CXCL9,-10,-11 and CCL-3L1, CCL-11 that were linked to NK cell recruitment (64, 111). Hence characterizing these NK cells would provide an in-depth understanding on their role in the lung mucosa and how they protect the lungs during a respiratory infection.

### Innate lymphoid cells

Innate lymphoid cells (ILCs) contribute to mucosal immunity and are found in several tissue sites, such as lungs, intestine, and skin (112, 113). Most ILCs are present in the respiratory tract and several types of ILCs that differ depending on their function, for instance, ILC-2 are associated with allergic airway inflammation and ILC-3 are associated with mucosal homeostasis (112, 114). During viral and bacterial pneumonia, ILC-3 released cytokines IL-17, IL-22 and IL-23 that led to microbial clearance and prevented tissue damage and inhibited secondary infection. ILC-3 cell-mediated lung immunity was found to involve other immune cells, including DCs that are associated with antigen presentation that leads to accumulation of IL-22-producing CCD6^+^ ILC-3’s in murine lungs (115, 116). During *K. pneumoniae* infection in mice, inflammatory monocytes are activated and recruited to the lungs, resulting in production of TNF-alpha and accumulation of CCR6^+^ ILC-3s in the lungs. Notably, the movement of ILC-3s from the circulation to the lungs during pneumonia was associated with CCR4 expression, as deficiency in this chemokine prevented cellular homing (117). In COVID-19, ILC-2 upregulated CCR10 cytokine expression which was linked to pneumonia recovery and inversely correlated with lung inflammation and lung injury markers IP-10, IL-18, IL-10, IL-15, IL-17, M-CSF, TGF-α, CXCL13 (113). Hence ILCs can be potential mediators of pulmonary recovery from respiratory infections.

## Circulating and tissue resident memory T cells

Human host protection against pathogens is highly reliant on the prevalence of immunological memory. Memory T cells play an important role in the recognition of antigens derived from both infectious pathogens and tumours. At birth, T cells are naïve regarding antigen exposure, with the expansion of the memory T cell pool developing over time. By ~20 years of age memory cells constitute more than 35% of circulating T cells (118, 119). Individuals under 10 years of age are more vulnerable to pathogens compared to in the following two decades of their life (53). By late adulthood, the adult T cell repertoire increasingly reflects a function of expansion and contraction of the existing memory T cell repertoire. In the later years of life, after 65 years, there is a reduction in the memory T cell production and function (120).

To fully unveil how memory T cells fight against infections, it is essential to appreciate the distribution of these cells in the body. Only 2-2.5% of the total and memory populations of T cells are present in peripheral blood (118). The remaining populations of memory T cells are localised in organs such as the lymph nodes, liver, spleen, lungs, skin, and the gut including the mucosal tissues and the lymphoid sites. When a respiratory pathogen such as influenza invades, the T cells are primed and activated in the lymphoid sites due to local antigen exposure and presentation. Antigen-specific CD4^+^ and CD8^+^ memory T cells proliferate, expand, and differentiate into effector memory T cells (T_EM_) that can migrate to tissue sites, and a subset of these express CD45RA (T_EMRA_) (118). T_EM_ cells can accumulate in numerous tissue sites such as the lungs and can be replenished from the lymphoid sites *via* circulating memory T cells.

A distinct subset of memory T cells that are developmentally and phenotypically distinct from circulating memory T cells called tissue resident memory T cells (T_RM_) reside in specific tissue sites and are known to generate a rapid localized immune response against respiratory pathogens. These cells are divergent from the circulating T cells and offer front line mucosal defence by expressing inflammatory and cytotoxic mediators. Therefore, it is critical to further understand their distinct role in the mucosal immunology of the lungs and associated BAL (121).

Inhaled respiratory pathogens make initial contact with the nasal cavity and upper respiratory tract therefore these sites may play a key role in inhibiting their spread to the lungs and other organs. Influenza-specific T_RM_ cells in the nasal cavity have the ability to inhibit the spread of influenza virus to the upper respiratory tract and consequently prevent severe pneumonia in mouse models (122). T_RM_ cells in nasal tissue or upper respiratory tract reside at higher numbers post infection compared to these cells in the lungs and have been associated with favourable prognosis (66, 122).

Little is understood about the generation and persistence of T_RM_ cells in human lungs. Snyder *et al.*, investigated the maintenance of T_RM_ cells in human BALs of lung transplant patients. Here donor and recipient derived T cells in human BALs revealed that the donor-derived “passenger” T_RM_ cells resided for more than a year in the grafted lung. These donor-derived T_RM_ cells expressed canonical gene signatures for CD69, CD103 and C49a as well as the cytokines IFN-γ and IL-17 together with high levels of the checkpoint molecule PD-1. In contrast, recipient-derived T cells that infiltrated the grafted lung gradually acquired a T_RM_ phenotype in the months following lung transplantation. Notably, the persistence of donor derived T_RM_ cells had a good correlation to low clinical events of lung injury, primary graft disfunction and acute cellular rejection. Therefore, surveillance of T_RM_ cell dynamics could be clinically informative to understand the quality of lung mucosal immunity in vulnerable lung transplant patients (123).

Studies on lung CD4^+^ and CD8^+^ T_RM_ cells post-infection provide an insight to the role of these cells in defence against respiratory pathogens. In a study on lung airways of influenza infected mice, a plethora of CD4^+^ T_RM_ cells were detected that were characterised by expression of CD69 and CD11a (124). The production of CD4^+^ T_RM_ cells post influenza infection, was maintained, and remained long term in the lungs of mice (125). A comparative human study of influenza, RSV and non-typeable haemophilus influenza (NTHi) demonstrated that antigen-specific CD4^+^ T_RM_ cells were in low proportion in peripheral blood as compared to the lungs (126). Another study revealed accumulated influenza specific CD4^+^ T cells with high IFN-γ levels in the infected lung tissue compared to peripheral blood (127). Notably, heterogenous populations of influenza-specific memory CD4^+^ T_RM_ cells in mice lungs was reported to clear the infection and increased survival, independent of CD8^+^ T cells or B cells. These type of T cells had a high expression of CD69 and CD11a and mediated protection against respiratory viruses (128). These data suggest that CD4^+^ T_RM_ cells may play a critical role in the local antiviral immune protection.

However, contradicting studies have demonstrated the accumulation of influenza and RSV-specific CD8^+^ T cells expressing the tissue-residency marker in mice lungs and not peripheral blood (129). The persistence of these T_RM_ cells in the lungs was dependent on the ongoing conversion from T_EM_ to T_RM_ cells. RSV and influenza specific T_RM_ cells had the ability to proliferate in the lungs upon antigen re-exposure, therefore assisting in immediate immunological protection against respiratory viruses (129, 130). Interestingly, the expression of CD103 was identified only in CD8^+^ T_RM_ cells specific for influenza and not in those for Epstein Barr Virus (EBV) or Cytomegalovirus (CMV) indicating pathogen-dependent heterogenicity (131). Another report on influenza infected human lung tissue from donors, demonstrated enrichment of influenza-specific CD8^+^ T_RM_ cells that resided in the lung tissue. These CD8^+^ T_RM_ cells also downregulated CD28 expression, suggesting TCR activation. This activation was largely seen in the lung tissue compared to peripheral blood. There was significantly lower levels of cytotoxicity markers CD107a and granzyme-B in CD8^+^ T_RM_ compared to the CD103^-^ CD69^-^ subset. Notably, the CD8^+^ CD103^-^ CD69^-^ subset was more likely to produce high levels of anti-inflammatory cytokines such as IFN- γ and TFN- α compared to CD8^+^ T_RM_ subsets (122). These studies illustrate heterogeneous role for CD4^+^ T_RM_ and/or CD8^+^ T_RM_ cells in localised mucosal immunity, which require further delineation.

Recently CD4^+^ T cells have shown to be associated with recruitment of CD8^+^ T cells to the lung, including CD8^+^ T_RM_ cells (132, 133). In the setting of RSV, epitope-specific CD4^+^ T cells were present in low proportions in the airways prior to infection. The frequency of these cells in the BALs was significantly higher than compared to blood. After infection, these antigen-specific CD4^+^ T cells were present in high proportions in the airways. The majority of these cells expressed CD69 and a subset co-expressed CD103 consistent with a T_RM_ cell phenotype (133). During infection, all RSV-specific CD4^+^ T cells displayed upregulated CCR5 and downregulated CD62L in both blood and BALs. Additionally, pro-inflammatory cytokines and chemokine receptors CXCL10 and CXCR3 positively correlated with activated CD4^+^ T cell recruitment in BALs (133). Moreover, CXCL10 and CXCR3 have previously been associated with protection against RSV-induced lung pathology in mice *via* a pathway involving both dendritic cells and CD8^+^ T cells (134).

A study of nasal swabs from acute, early recovery and convalescent COVID-19 patients was conducted to determine if long-lived protective CD8^+^ T cell immunity develops in the nasal mucosa. The results demonstrated the presence of SARS-CoV-2-specific CD8^+^ T_RM_ cells with high expression of CD69 marker and low expression of KLRG1. Single cell RNASeq data, found differences in CD8^+^ T_RM_ cell clusters in acute, early recovery and convalescent patients. For instance, HLA-DR, CD38 and Tbet expression was higher in convalescent hospitalized patients compared to acute or early recovery patients. CD8^+^ T_RM_ cells with activated transcription profiles persisted 5-6 weeks after discharge from the hospital. After 1-3 months post viral clearance up to 70% of CD8^+^ T cells in the nasal passage had an activated T_RM_ cell phenotype in convalescent patients (135). Although this study provided important data on the prevalence of CD8^+^ T_RM_ in nasal swabs, it remains unknown if analogous populations exist in other parts of the upper respiratory tract.

Single cell RNA sequencing on BAL samples from patients with COVID-19 pneumonia demonstrated that lung T cells mainly expressed a T_RM_ phenotype and with evidence of associated clonal expansion (64). SARS-CoV-2-specific CD4^+^ and CD8^+^ T_RM_ cells were present in the lungs up to 10 months post infection, and the transcriptome of these cells differed from peripheral blood T cells. For example, during acute infection, IL-4 was secreted by SARS-CoV-2-specific T cells in hospitalized patients and IL-10 in non-hospitalized patients. In contrast (64), another study reported IL-10 production during active disease. High levels of IL-4, IL-10 and IFN-γ have been found in serum from patients with COVID-19 (136). Interestingly, IL-4 associated with CD4^+^ and CD8^+^ T_RM_ cells has been identified in lungs of patients that died due to COVID-19 (136, 137). The finding that IL-4 associated with severe COVID-19 and associated morality is potentially of high importance and warrants further investigation.

There is limited literature on the impact of senescence on the abundance of T_RM_ cells in the lungs. Although it is well established that CD8^+^ T cells in the lungs reduce in percentage, function and TCR diversity with age in mice (138). To investigate the influence of aging on T_RM_ cells, a study on T cell compartmentalization revealed that there was a higher proportion of CD8^+^ T_RM_ cells in human adults compared to youth but notably these numbers declined in older populations over time (139). Interestingly, aged mice after influenza infection had a higher percentage of dysfunctional influenza specific CD8^+^ T_RM_ cells compared to juvenile mice. From single cell data these T_RM_ cells in aged mice had lower effector functions against major influenza epitopes in response to TCR signalling (140). Another study in older mice model of SARS-CoV-2 infection reported the recruitment of CD8^+^ T_RM_ cells in the lungs but these cells did not provide any protective immunity to secondary infections and was linked to chronic lung disease. Moreover, these aged CD8^+^ T_RM_ cells were associated with increased tissue inflammation and lung collagen deposition (141). These studies revealed the role of age in CD8^+^ T_RM_ cells and associated disease progress, however, there is an urgent need to further understand T_RM_ dysfunction and pathogenicity for targeted therapy earlier on to ensure better patient outcome in viral pneumonia patients.

### Vaccine induced T_RM_ cells

An influential factor in the distribution of T_RM_ cells is the route and site of vaccine delivery. Intranasal administration of attenuated influenza viral vaccine increased the levels of CD4^+^ T_RM_ cells in lungs of mice that resulted in long-term protection against influenza variants (142). Vaccine-induced T_RM_ cells are reportedly not restricted to the location of immunization and can be identified in other distant mucosal sites such as the vaginal mucosa (143). In contrast, a study of intranasal immunization against influenza A virus reported T_RM_ cells remained embedded in the large airways and walls of the lungs in mice, but this is absent in systemically primed mice (144). Previous studies on mice have revealed that lung CD4^+^ and CD8^+^ T cells increase along with cytokine production after influenza restimulation or vaccine administration (142, 145, 146). Vaccine strategies for influenza include tetravalent inactivated influenza vaccine (TIV) or live attenuated influenza vaccine (LAIV). These vaccines can be administered intramuscularly or *via* the nasal passage. Nasal administration of LAIV elicited a localized immune response in the lungs and nose with high levels of CD4^+^ T_RM_ cells and virus-specific CD8^+^ T cells. Virus-specific CD4^+^ T_RM_ cells expressed high levels of IFN-γ and TNF-α cytokines and CD8^+^ T cells expressed high levels of TNF-α. Interestingly, when LAIV was administered, there was a significant reduction in the colonization of *S. pneumoniae* in the nasopharynx and this was not observed when TIV was administered intramuscularly (146). Hence this demonstrated that vaccine strategy and lung T_RM_ cells may play a role in more broadly protective immunity.

## The lung microbiome

The lung microbiome protects against pneumonia and other respiratory infections by preventing the colonization of pathogenic bacteria or viruses and by regulating the immune response (147). Microbial colonies present on the mucosal surfaces of the respiratory tract shape the immune responses and influence the relationship between the host and pathogens (148). The lung microbiome is comprised of a multitude of species, most of these being commensals derived from the oral cavity (149). One report suggests that the lung and oral microbiome are indistinguishable, as sequencing of the microbiome demonstrated that the oral cavity and the lung microbiome carry comparable bacterial commensals. The oral and lung microbiome therefore may be considered as one entity and poor oral hygiene has been found to be a major contributing factor for pathogenic microbes entering the upper respiratory tract. For instance, dental plaques result in biofilm formation on the teeth harbouring pathogenic bacteria that can then seed and infect the lungs, resulting in respiratory disease (150, 151).

Critically ill patients in the ICU can rapidly accumulate dental plaques that become colonised by Gram-negative or aerobic bacteria. These oral pathogens can become incorporated into the lung microbiome, resulting in alterations of healthy epithelial cells including the loss of fibronectin that facilitates the adhesion of pathogenic *P. aeroginosa* (152). BAL samples taken from the lower respiratory tract of individuals that harboured oral microbes such as *Prevotella* spp., and *Veillonella* spp., contained an increase in lymphocytes and neutrophils and evidence of Th17 inflammation compared to healthy individuals (153). This suggested that there may be movement of microbes between the oral cavity and the lung or upper respiratory tract. However, there is no evidence to-date regarding how this affects the pathogenesis of inflammatory lung disease (154).

The lung microbiome is typically altered in patients with a variety of respiratory diseases, as compared to healthy individuals (155). A study of mice with ARDS showed that the lung microbiome is altered as early as 5 days post-sepsis as compared to controls. It was also demonstrated that the lung microbiome was enriched with viable gut-derived bacteria (156). This altered lung microbiome had high concentrations of cytokines TNF-α, MIP-1β and G-CSF in the BAL samples that positively correlated with alveolar inflammation (156).

A major factor that influences the composition of the lung microbiome is the use of antimicrobial therapies. A cohort on ARDS patients exposed to antimicrobial therapy, such as cephalosporins, penicillins, carbapenems and sulphonamide’s harboured an over-representation of bacteria belonging to antimicrobial resistant *Enterobacteriaceae* in the lung microbiome (157). ICU patients treated with decontamination of digestive tract, also had high prevalence of drug resistant *Enterobacteriaceae* (156), implying that the lung microbiome of ARDS patients commonly harbours this family of bacteria independent of the gastrointestinal tract. Such data suggests that pulmonary dysbiosis may play a role in propagating a hyperinflammatory immune response (150).

Dysbiosis in the lung microbiota during COVID-19 pneumonia and the presence of specific pathogens in the lung microbiome can be correlated to increased risk of disease progression and mortality (158). Genomic sequencing of BAL samples from patients with COVID-19 pneumonia found an abundance of pathogens including *Acinetobacter* spp., *Pseudomonas alcaligenes*, *Clostridium hiranonis* and *Enterobacteriaceae* in the lungs compared to those with non-COVID-19 pneumonia. Studies of other respiratory infections, such as RSV harboured abundant influenza species, in patient BAL samples (159, 160). In the non-COVID-19 patients that were in the ICU, there was a less diverse range of pathogens, such as *Haemophilus influenza, Streptococcus* and *Veillonella* spp (158). Oral-lung microbiome and associated diversity can disrupt the microbiome homeostasis during respiratory infection and influence susceptibility and disease severity (161, 162). Using alpha diversity of Faiths phylogenetic index, it has been shown that in COVID-19 patients the oral microbial diversity was lesser compared to healthy controls. Notably, significant reduction was mainly observed in severe COVID-19 patients rather than patients with mild COVID-19. Another diversity index called the criteria-individual beta diversity index indicated that the microbiome from both severe COVID-19 patients and mild COVID-19 clustered separately to healthy controls, suggesting that the microbial content differs depending on COVID-19 disease severity. Microbial biomarkers were also examined, where 17 divergent taxa were detected between healthy and COVID-19 oral microbiome (161). Together, these findings suggest that the dysbiosis of the respiratory microbiome contributes to the pathogenesis of respiratory disease.

The lung microbiome has been correlated to infectivity and clinical outcomes in pneumonia and alterations in the lung microbiome have been correlated to high mortality (163). To explore the functional role of lung microbiome in COVID-19 pathogenesis and how this may alter metabolic pathways, a meta transcriptomic study was conducted on 8 COVID-19, 25 CAP and 20 healthy controls (164). Metabolic pathway mapping revealed pathways that were reduced in COVID-19 patents that included: glycan biosynthesis, glycan degradation and lipid metabolism and sphingolipid metabolism. Spingolipids are a part of the biomembrane and are involved in signal transduction and immune activation. Interestingly, spingolipids were not only reduced in BALs but have been reportedly lower in serum of COVID-19 patients (165), indicating they may play a role in immune dysfunction. Functional features associated with these pathways were investigated and demonstrated that 3.2.1.22 alpha galactosidase (alpha-gal) was lower in COVID-19 patients, which was linked to sphingolipid metabolism and glycerolipid metabolism. Higher alpha-gal titres in the microbiome of individuals were linked to reduced severity of COVID-19. The metabolic pathways that were upregulated in COVID-19 patients included the carbohydrate metabolism like glycolysis and gluconeogenesis. Previous study on faecal samples of COVID-19 patients demonstrated that high carbohydrate metabolism was linked to high infectivity (166). These studies therefore characterized the respiratory microbiome in COVID-19 patients however due to their limitation of sample size and lack of clinical data points these findings require further validation.

## Acute vs long COVID-19

Acute COVID-19 disease is characterised by varied clinical manifestations ranging from asymptomatic to severe. After the acute symptomatic infection, these symptoms may persist longer than 3 months from the time of infection. This is known as ‘long COVID’ or post-acute sequelae of COVID-19 (167). A UK report recorded patients with symptomatic COVID-19 infection, that developed long COVID after 4 weeks, 8 weeks and 12 weeks were 14%, 5% and 2% of the total cohort, respectively (168). Common symptoms include fatigue, dyspnea, headache, anosmia, and these symptoms are frequent in older patients. Long COVID is prevalent in 10-30% of unvaccinated community-managed COVID-19 patients, 2-3 months post infection. In 76% of patients that initially required hospitalisation, the symptoms persisted for 6 months (169, 170). Another UK study recently published on the likelihood of long COVID in delta and omicron cases demonstrated that omicron variant is less severe with a reduced associated risk of long COVID compared to the delta variant. Overall, 4.5% of omicron cases experienced long COVID compared to 10.8% of delta cases. Unvaccinated patients that acquire SARS-CoV-2 infection showed a higher risk of long COVID and death compared to vaccine-breakthrough infection (171). Populations vaccinated with the BNT162b2 and mRNA-1273 vaccines had a 15% lesser risk of acquiring long COVID (172).

A recent report on an Australian cohort found the majority of long COVID patients had elevated activated T cells and B cells and a low proportion of naïve cells (173). To investigate biomarkers associated with long COVID, 28 serum analytes from patients 4 months post infection were measured. Six inflammatory cytokines: IFN-ß, type III IFN, CXCL-9, CXCL-19, IL-8 and soluble T cell immunoglobulin mucin domain 3 (sTIM-3) that are generally expressed by monocytes, macrophages and memory T cells were present in high levels in long COVID patient compared to controls. The levels of IFN-ß, type III IFN remained high until 8 months post infection in the serum. This study defined the immunological markers associated with long COVID in peripheral blood (173), and further work in this area aims to determine if these changes are related to ongoing antigen persistence.

SARS-CoV-2 infections can lead to long-term immunological memory as SARS-CoV-2 specific antibodies and T cells are prevalent even after 8 months of infection (174). However, the localised immunological landscape, within the lungs, during long COVID or after recovery is not well understood. One study explored the immune landscape in the respiratory tract during long COVID (175). They investigated BAL and blood samples from patients with respiratory abnormalities 3-6 months post COVID-19 infection. Significantly high number of macrophages, NK, T cells and B cells were present in the airways as compared to blood. In long COVID patients, proportions of monocytes and neutrophils were lower in BALs compared to blood, indicating that maybe these cells are associated with acute COVID-19 infections and not long COVID. Expression of chemokine receptors CXCL9 and CXCL11 positively correlated to the lymphocyte and T cell counts. An increase in total IgG levels correlated to increase in B cell count in BALs. The same group also investigated the proteomic signatures in the airways to reveal that 22 proteins that were involved in leukocyte activation, cell death and response to injury were upregulated. When respiratory airways were assessed after 1 year of COVID-19, their T cell, B cell, NK and neutrophil, AM count decreased significantly. The reduction in the infiltration of immune cells increased the risk of acquiring pneumonia (175). Hence this study demonstrated the dynamic nature of the immune landscape. This study raises important questions around the long-term sequalae of COVID-19, however it should be noted that the study had a relatively small sample size (N=38) and did not have any baseline pre-infection samples to exclude baseline confounders.

## Local immune response in COVID-19 pneumonia

The current SARS-CoV-2 pandemic has resulted in a sudden increase in the incidence of severe CAP (176) and related morbidity and mortality from COVID-19. The SARS-CoV-2 virus initially propagates while instigating a limited innate immune response, however once it reaches the lower respiratory tract, the virus triggers a far more robust innate response (177, 178). If SARS-CoV-2 infection persists it can result in lung damage, pneumonitis, ARDS and/or sepsis and is commonly accompanied by profound dyspnoea and hypoxic respiratory failure (179).

The immunological response to SARS-CoV-2 has largely been characterised from the peripheral blood, with perturbations in both the innate and adaptive immune systems observed. When a healthy individual is infected by SARS-CoV-2 the virus stalls the innate immune response and delays the release of IFN type I and type II. This results in the virus easily replicating due to lack of adaptive immune response. This lag in the innate immune response results in non-symptomatic infection (180).

Autoantibodies against type I interferon (IFN) are a risk factor in severe viral respiratory disease. Type I IFN are ubiquitously expressed cytokines that contribute to innate, and cell mediated immunity against viruses. Notably, autoantibodies neutralise 13 different IFN-α and 1 IFN-ω, but rarely neutralise IFN-β, κ and ϵ during viral infections (181). A landmark study found that 10% of COVID-19 pneumonia patients produced autoantibodies which neutralised IFN-α and IFN-ω, 37% of these patients did not survive. These neutralising autoantibodies were absent in patients with asymptomatic or mild COVID-19 infection as well as healthy controls (181). Another report found 13.7% of severe COVID-19 pneumonia patients had IgG autoantibodies against at least one type of type I IFN. Moreover, the study revealed that neutralising autoantibodies were identified in severe COVID-19 patients with complications, in 33% patients with bacterial pneumonia, 67% with thromboembolic events and all the ICU patients associated with high mortality (182). Hence autoantibodies against type I IFN have been reported to be triggered after SAR-CoV-2 infection in patients, implying that the production of IFN due to the virus may be crucial for shielding against severe disease. The presence of neutralising antibodies against these IFNs may serve as an advantage to the virus, consequently limiting the innate and adaptive immune responses. The inadequate production of type I IFN associated with IgG antibodies during the initial stages of COVID-19 is likely to result in viral dissemination to the lungs *via* the bloodstream (183, 184). IgG antibodies have been implicated in exacerbating the pathology of SARS-CoV-2 infections by increasing the production of pro-inflammatory IL-8 and CCL2 by macrophages leading to disruption of the epithelial barrier and thrombosis (185, 186). High anti-spike IgG and reduced fucosylation was linked to inflammation due to increased affinity to FcγRIII receptor. This FcγRIII receptors were found in monocytes and neutrophils in inflamed lungs (187). These studies highlight the contribution of pathogenic IgG antibody in inflammation during severe COVID-19. This inflammatory mechanism could be potentially useful as target for treatment of severe COVID-19.

**Figure 1:**
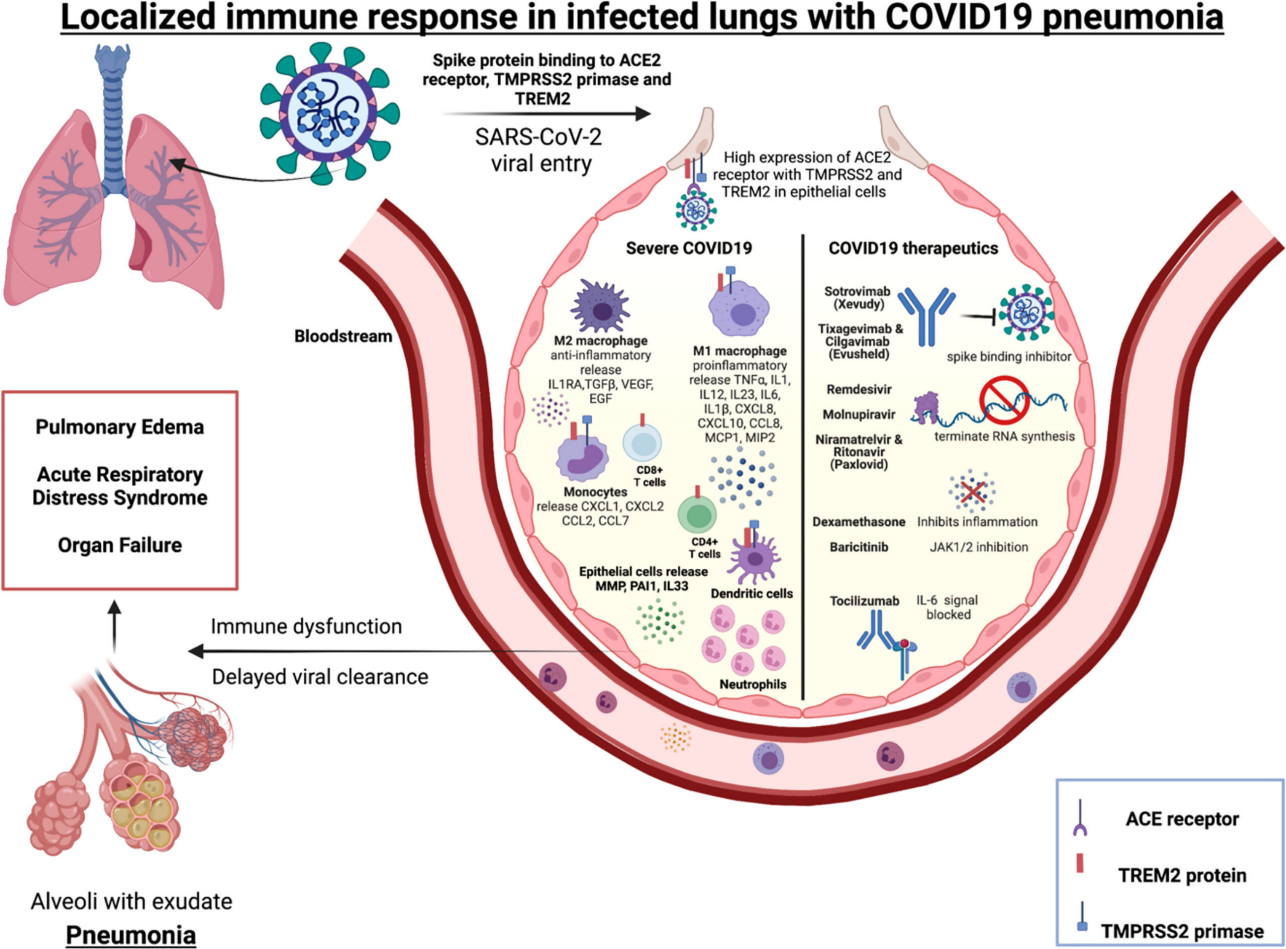
Immune mediators of severe COVID-19 and targets for licensed therapeutics.*Alveolus left side:* Immune cells (macrophages, monocytes, T cells, neutrophils and dendritic cells) localized in the lungs release specific cytokines and chemokines along with high neutrophil to lymphocyte ratio (NLR) during severe COVID-19 pneumonitis. *Alveolus right side:* Molecular mechanism of action of COVID-19 therapeutics include blocking the spike binding receptor (Xevudy), terminating viral RNA synthesis (Remdesvir, Niramatrelvir, Ritonavir, Molnupiravir). Corticosteroids (Dexamethasone) block transcription of glucocorticoid-responsive genes, many of which are pro-inflammatory e.g. Type 1 interferons. Janus kinase (JAK) inhibitors that block the signalling pathway (Baricitinab). IL-6 signalling can also be targeted (Tocilizumab).

In severe COVID-19, low number of SARS-CoV-2 antigen-specific CD4^+^ and CD8^+^ T cells have been reported in BALs along with increase in inflammatory monocytes and macrophages (**Figure 1**) compared to mild COVID-19 (188). Activated monocytes have been reported to migrate to the lungs in severe COVID-19 infection (188). Increased IL-6, MCP-1 and IL-10 have been detected in COVID-19 BALs compared to peripheral blood (189). Other studies of blood demonstrated that severe COVID-19 is associated with an increase in IL-6, activated macrophages and neutrophils and a T cell lymphopenia that contribute to lower proportions of antigen-specific CD4^+^ and CD8^+^ T cells (64, 190–192).

SARS-CoV-2 activates the lung epithelial pro-inflammatory signals on entry, leading to severe mucosal inflammation and airway damage (102). This may be due to entry-associated receptor ACE2, TREM-2 protein and protease enzyme TMPRSS2 that are expressed on a broad range of immune cells, including macrophages, dendritic cells, and monocytes. The SARS-CoV-2 receptor ACE2 and priming enzyme TMPRSS2 is highly expressed in lung epithelial and bronchial cells and this positively correlated with interferon and other proinflammatory signals (193). A study of patients with severe COVID-19 found hyperactivated epithelial lung cells expressed matrix metalloproteases (MMP) and plasminogen activation inhibitor-1 (PAI-1), and there was decreased IFNA1 that contributed to a dysregulated pulmonary immune response (194). Therefore, there is evidence that SARS-CoV-2 can modify the lung epithelium and immune cells in severe cases. In macrophages derived from patients with COVID-19, high levels of TREM-2 and TMPRSS2 mRNA, was associated with higher hypoxia scores, implying poor overall survival of these patients (195). In severe COVID-19 disease the soluble mediators IL6, IL1β, CXCL8 and CXCL10 are expressed in a TREM-2 dependent manner by macrophages (196). Other studies have demonstrated that TREM-2 is constitutively expressed on T cells during SARS-CoV-2 infection leading to rise in IL-2, IFN-γ, IL-1B and IL-4 thereby being a key mediator in T cell immunity and inflammation (197). TREM-2 expression was higher in peripheral blood and lung infiltrating CD4^+^ and CD8^+^ T cells of severe and non-severe COVID 19 patients than healthy donors. TREM-2^+^CD4^+^ T cells was significantly high in lung tissue of non-severe COVID19 patients. The study also examined the production of granzyme B, an intracellular cytotoxic protease. Its level was increased in TREM-2 expressing CD4^+^ T cells and blockage of TREM-2 signals with TREM-2-Fc fusion protein consequently reduced expression of granzyme B. TREM-2^+^CD4^+^ T cells were associated with indicators of severe COVID-19 pneumonia, namely CRP and lymphocyte count. Immunofluorescence microscopy was conducted to reveal the localisation of TREM-2 in CD4^+^ T cells in lung tissue of non-severe COVID-19 patients. Hence TREM-2 may be involved in lung localised CD4^+^ T cells in COVID-19 patients. TREM-2 expression on both CD4^+^ and CD8^+^ T cells was correlated to TCR dependent markers including CD134 (OX-40), CD137, CD69 and CD25 (197). This implies that TREM-2 plays critical role in T cell activation and differentiation in SARS-CoV-2 pneumonia. In summary these studies demonstrate that SARS-CoV-2 entry-associated protein TREM-2 is expressed in several immune cells may be crucial to mount a localised immune response in the diseased lungs.

To further explore the lung microenvironment in COVID-19, techniques such as single cell RNA sequencing of BAL fluid offer detailed snapshot of the lung immune cell heterogeneity in patients that have varying severity. Transcriptional analysis unveiled there are differences observed in the cytokine profiles of BAL compared to PBMCs (198). Specifically, pro-inflammatory molecules CCL2, CCL8 that are chemokine CC motif ligands typically expressed on monocytes, memory T cells and dendritic cells and CXCL1, CXCL2, expressed on monocytes and macrophages and interleukin IL-33 expressed by epithelial lung cells were found in severe COVID BAL fluid and CXCL10, TNFSF10, TIMP1, IL-10, IL-18, AREG, NRG1 was present in the PBMCs, indicating inflammatory signature pathways. Higher numbers of effector memory like CD4^+^ and CD8^+^ cells, CD14^+^CD16^+^ monocytes, neutrophils were observed in respiratory samples of COVID-19 patients compared to the levels found in peripheral blood (179, 199). Respiratory tract sampling from patients with COVID-19 found high levels of IL-6, IL-8 and MCP-1 (200). Another study on SARS-CoV-2 pneumonia where single cell data on BALs revealed that the airways were rich with CD8^+^ and CD4^+^ T cells and monocytes. Interestingly, SARS-CoV-2 was detected in AMs in 68% of BALs. AMs contributed to IFN-γ production from activated T cells. Two different clusters of tissue resident AM were observed in BALs, one of which was infected and other uninfected. The infected AM cluster expressed cytokines and chemokines like CCL4, CCL20, CXCL10 and CCL11 associated with monocytes and T cell recruitment in the lungs (201). Such studies illustrate the divergent transcriptional profiles between the respiratory samples and peripheral blood in patient with COVID-19.

Along with T cells, B cells are also known to play a critical role in adaptive immunity against SARS-CoV-2 infection. In peripheral blood, varied B cell phenotypes have been found to be associated with the severity of COVID-19 (194). In a study of patients with severe COVID-19, B cells expressed high levels of immunoglobulins along with abundant CCR2^+^ plasma cells. Prominent levels of CD25, IL-8,IL-9 and IL-10 were measured in B cells from peripheral blood of severe COVID-19 patients (202). SARS-CoV-2 virus-specific B cell responses along with CD4^+^ T cell responses have been detected in peripheral blood 1 week from the onset of symptomology. After 2-3 weeks post infection, neutralising antibodies (NAbs) that were specific towards receptor binding domain (RBD) in the spike protein released. The majority of studies on COVID-19 patients studied <4 months from infection demonstrated that these NAbs are short-lived and reduce after 1-2 months (203). However, there are no studies so far on NAbs that are associated with COVID-19 in BALs. B cells producing SARS-CoV-2 specific IgA antibodies has been identified 1 week post infection in BALs and these IgA secreting B cells were found to reside in the lung mucosa (204).

Limited studies have been conducted on BAL to explore the role of B cells and NAbs in localized immunity in COVID-19 patients. A recent study on transcriptomic analysis of COVID-19 BAL samples reported higher relative proportion of B cells compared to other immune cells. In contrast, another flow cytometric study of COVID-19 BAL samples demonstrated that the lymphocytic subsets comprised of very low, ~ 0.9% of B cells and high percentage, ~86% of T cells (205). The literature on COVID-19 predominately focuses on B cells in peripheral blood rather than in BAL. Hence there is no clear understanding on the role of these immune cells in localised immune response in the lungs, which is the main site of infection and damage in SARS-CoV-2 infection.

### COVID-19 therapeutics

The current licensed therapeutics for COVID-19 highlight the multifaceted immunological response to SARS-CoV-2, and opportunities for intervention (**Figure 1**). These therapeutics include monoclonal antibodies that prohibit the virus from binding its receptor (e.g. Sotrovimab, Evushield) (206, 207); other therapeutics incorporate into new viral RNA but prevents ongoing synthesis (eg: Remdesvir, Molnupiravir, Paxlovid) (208). Therapeutics also include broad spectrum immunosuppressives such as dexamethasone, that has broad anti-inflammatory and immunosuppressive effects. Other inhibitors that target specific signalling pathways (e.g. JAK inhibitor Baricitinib) and IL-6 signalling inhibition (Tocilizumab) leading to anti-inflammation. In summary, SARS-CoV-2 therapeutics target specific receptors, or ligands for viral entry, replication, protein synthesis or inhibit release of inflammatory signals and are likely to be involved in triggering the localized immunity.

## Discussion

CAP continues to have a high morbidity and mortality burden worldwide, despite continual advancements in the development of advanced life support and antimicrobials. CAP can be a precursor to sepsis, which remains a current global health priority. In developed countries part of the high burden of CAP may relate to the significant effects of an aging population, as advanced age has been clearly replicated to be a major risk factor for the development of severe CAP (207).

In the hospital setting, clinical monitoring of pneumonia currently involves measurement of observations such as temperature, heartrate, BP, respiratory rate and SpO2, lymphopenia together with serial chest x-ray and peripheral biomarkers such as CRP and procalcitonin (209). There is a growing acknowledgement that the molecular and cellular pathogenic processes that occur in the frontline of infected tissues may not always be discernible through the sampling of peripheral blood. A greater understanding of the complexity of pulmonary mucosal immunity, including the barrier functions of the lung epithelium and interactions between innate and adaptive (including tissue-resident populations) and their communications, response to soluble mediators and cytokines will increase our understanding of the pathogenesis of CAP to determine correlates of successful recovery versus those that signify a poorer prognosis.

There is much work to be done on how these cellular and molecular components interface with additional host factors such as genetics and the microbiome. The astounding medical and scientific advancements on our understanding of COVID-19 in such a short period of time, and particularly the proven efficacy of host-directed immunomodulators such as Dexamethasone, Baricitinib and Tocilizumab have revealed an opportunity for advancements made in the current pandemic to be further explored and potentially translated into the care of patients with CAP and respiratory sepsis more generally.

## Author contributions

SS and PH collated the relevant literature, wrote the initial drafts and designed the figure. CA and AK revised the article and provided additional expertise. All authors reviewed and approved the final article.

## Conflict of interest

The authors declare that the research was conducted in the absence of any commercial or financial relationships that could be construed as a potential conflict of interest.

## Publisher’s note

All claims expressed in this article are solely those of the authors and do not necessarily represent those of their affiliated organizations, or those of the publisher, the editors and the reviewers. Any product that may be evaluated in this article, or claim that may be made by its manufacturer, is not guaranteed or endorsed by the publisher.
